# Supplementary material for: Causal Relationship between Aging and Anorexia Nervosa: A White-Matter-Microstructure-Mediated Mendelian Randomization Analysis
Source: Biomedicines. 2024 Aug 16;12(8):1874. doi: 10.3390/biomedicines12081874 (PMC11351342; doi:10.3390/biomedicines12081874)
Supplement: Supplementary file 1 [file biomedicines-12-01874-s001.zip › Supplementary Figure/Figure S4/Singe SNP plot of MR analysis for TL to ubm-b-2122.pdf]

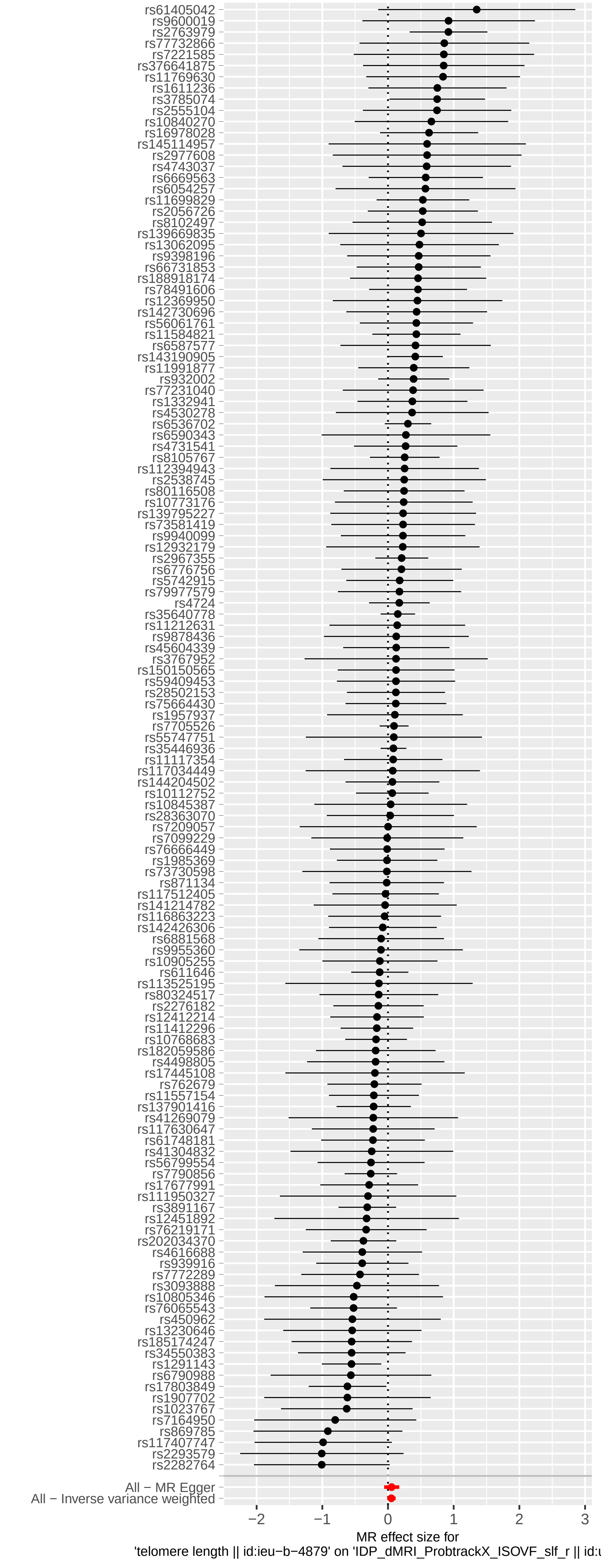
rs61405042
rs9600019
rs2763979
rs77732866
rs7221585
rs376641875
rs11769630
rs1611236
rs3785074
rs2555104
rs10840270
rs16978028
rs145114957
rs2977608
rs4743037
rs6669563
rs6054257
rs11699829
rs2056726
rs8102497
rs139669835
rs13062095
rs9398196
rs66731853
rs188918174
rs78491606
rs12369950
rs142730696
rs56061761
rs11584821
rs6587577
rs143190905
rs11991877
rs932002
rs77231040
rs1332941
rs4530278
rs6536702
rs6590343
rs4731541
rs8105767
rs112394943
rs2538745
rs80116508
rs10773176
rs139795227
rs73581419
rs9940099
rs12932179
rs2967355
rs6776756
rs5742915
rs79977579
rs4724
rs35640778
rs11212631
rs9878436
rs45604339
rs3767952
rs150150565
rs59409453
rs28502153
rs75664430
rs1957937
rs7705526
rs55747751
rs35446936
rs11117354
rs117034449
rs144204502
rs10112752
rs10845387
rs28363070
rs7209057
rs7099229
rs76666449
rs1985369
rs73730598
rs871134
rs117512405
rs141214782
rs116863223
rs142426306
rs6881568
rs9955360
rs10905255
rs611646
rs113525195
rs80324517
rs2276182
rs12412214
rs11412296
rs10768683
rs182059586
rs4498805
rs17445108
rs762679
rs11557154
rs137901416
rs41269079
rs117630647
rs61748181
rs41304832
rs56799554
rs7790856
rs17677991
rs111950327
rs3891167
rs12451892
rs76219171
rs202034370
rs4616688
rs939916
rs7772289
rs3093888
rs10805346
rs76065543
rs450962
rs13230646
rs185174247
rs34550383
rs1291143
rs6790988
rs17803849
rs1907702
rs1023767
rs7164950
rs869785
rs117407747
rs2293579
rs2282764
All − MR Egger
All − Inverse variance weighted
−2
−1
0
1
2
3
MR effect size for
'telomere length || id:ieu−b−4879' on 'IDP_dMRI_ProbtrackX_ISOVF_slf_r || id:u
